# Nursing and midwifery in a changing world: Addressing planetary health and digital literacy through a global curriculum

**DOI:** 10.1002/nop2.2075

**Published:** 2023-12-23

**Authors:** Ken Hok Man Ho, Ho Yu Cheng, Lisa McKenna, Daphne Sze Ki Cheung

**Affiliations:** ^1^ The Nethersole School of Nursing, Faculty of Medicine The Chinese University of Hong Kong, Shatin, New Territories Hong Kong, SAR China; ^2^ School of Nursing and Midwifery La Trobe University Melbourne Victoria Australia; ^3^ School of Nursing The Hong Kong Polytechnic University Hong Kong, SAR China

In May 2023, the World Health Organization declared an end to COVID‐19 as a global health emergency. The COVID‐19 pandemic exacerbated shortages of nursing and midwifery workforces and promoted their mobilization and migration globally. Since nurses and midwives play a critical role in health promotion, disease prevention and delivery of health care, equipping them with planetary health and digital literacy is crucial for a responsive workforce in the 21st century.

In this editorial, we discuss the importance of planetary health and digital literacy in nursing and midwifery education in an era with high nursing and midwifery mobility. We aim to raise further discussion among stakeholders on the potential of global curricula for the education of nurses and midwives internationally.

## 1 HIGH NURSING AND MIDWIFERY MOBILITY

Global nursing and midwifery workforce shortage is a critical issue to meet the demands of population health. According to the World Health Organization (2021a), the global nursing workforce of 27.9 million accounts for a needs‐based shortage of 5.9 million nurses. Almost 90% of the global nursing shortage is in low‐ and lower‐middle‐income countries. In contrast, about 70% of the projected increase in nursing workforce will occur in upper‐middle and high‐income countries by 2030. A similar picture of shortage of midwifery workforce is seen in low‐ and lower‐middle countries. The global nursing and midwifery workforce accounts for nearly 50% of the global health workforce. Meanwhile, the global shortages of nursing and midwifery also account for 50% of the current shortage in health workers (World Health Organization, 2021a).

Exacerbated workforce shortages after the COVID‐19 pandemic have resulted in negative consequences on population health, such as cessation of non‐critical healthcare services and inadequate coverage of health services in rural areas. To alleviate negative impacts of manpower shortage, various developed countries, such as Australia and Canada, highly prioritized nurses and midwives in their wanted skills lists for immigration. Globally, one in eight nurses migrate to work in another country where they were not born or educated (World Health Organization, 2021a). In most cases, foreign‐educated nurses coming from low‐ and lower‐middle‐income countries moved to high‐income countries for higher wages. It was suggested that international nursing and midwifery workforce mobility and migration actually further increased after the COVID‐19 pandemic because of greater demands for workforce from high‐income countries, as well as relaxed barriers to international travel or entry into practice (World Health Organization, 2021a).

The international migration of nurses and midwives implies there is an increasing diversity among the workforce. For example, the nursing and midwifery curricula of the origin countries may only satisfy the domestic needs of the origin countries. Although foreign‐educated nurses need to fulfil the registration requirement of host countries, the content of the examination is usually still country‐specific. A more comprehensive global education programme is essential to prepare the nurses for working both domestically and internationally.

## 2 PLANETARY HEALTH

The health of our planet is intricately linked to human well‐being, and it is crucial that nurses and midwives recognize the urgent need to address climate change. Climate change has been identified as one of the most statistically significant health challenges we face in the 21st century (Campbell‐Lendrum et al., 2023). It is not only responsible for more than one‐third of heat‐related deaths and proliferation of infectious diseases, but also contributes to the rise of non‐communicable diseases, including mental disorders (Vicedo‐Cabrera, 2021). In addition, the choices we make in terms of development and economy directly impact the climate crisis, which in turn affects our health reciprocally. For instance, urbanization often contributes to global warming and physical inactivity, leading to various health burdens. Therefore, we suggest that planetary health should be incorporated as a core component of nursing and midwifery education, research and practice.

Planetary health encompasses both the well‐being of human civilization and the state of the natural systems on which our survival depends. In order to effectively adopt a planetary health perspective, nurses and midwives worldwide require support in reforming educational curricula to integrate environmental and climate change knowledge. It is vital that they engage in research that establishes connections between the climate crisis and the nursing and midwifery profession. Furthermore, the workforce should develop advocacy skills to promote climate‐conscious policies within the healthcare sector. Considering that the healthcare sector itself accounts for 4.4% of global emissions (Campbell‐Lendrum et al., 2023), nurses and midwives must be prepared to adopt planetary health perspectives. The need to address this issue is imminent, and it is crucial that we equip the workforce with the necessary knowledge and skills to mitigate the impact of climate change on human health.

## 3 DIGITAL LITERACY

The World Health Organization emphasizes the immense potential of digital technology in achieving universal health coverage and transforming health outcomes, provided it is implemented correctly (World Health Organization, 2021b). To promote equitable, affordable and universal access to healthcare benefits, it is crucial to cultivate a digitally capable nursing and midwifery workforce. Digital health surpasses the scope of eHealth and encompasses a range of digital technologies, including robotics, advanced computing, big data analytics and artificial intelligence (AI). Preparing nurses and midwives to be digitally literate does not require in‐depth understanding of the intricacies of each digital technology. Instead, it involves equipping the workforce with the skills to actively engage with digital health technologies in care delivery, fostering mindsets of continuous learning about these technologies and cultivating abilities to critically evaluate information generated by AI. As the nursing and midwifery profession evolves in the era of digital health, they are expected to assume diverse roles and adapt to new care delivery models. This necessitates the transformation of their core competencies and educational requirements. Nurses and midwives must actively contribute to, and keep pace with, the digital transformation, ensuring that digital health technologies are developed and applied in a manner that aligns with the needs of individuals, families, communities and the broader healthcare workforce (World Health Organization, 2021b). By embracing digital health, nurses and midwives can seize opportunities to improve healthcare delivery, enhance patient outcomes and drive positive change in the healthcare landscape without the geographical boundary.

## 4 IMPLICATIONS FOR NURSING AND MIDWIFERY CURRICULA

The increasingly global nature of nursing and midwifery, along with the global challenges facing these professions, raises the question of whether current curricula are fully fit for purpose. Curricula leading to registration as nurses or midwives are generally accredited by local registering authorities in a single country and accreditation criteria needing to be met align with local practice standards and healthcare delivery systems. There may also be specific local health issues that further influence nuances of curricula in certain countries. How curriculum standards are developed likely varies greatly from country to country, and are potentially influenced by practitioners and policy makers in the particular country. Whether there is international involvement in standards development is unclear, and this aspect may currently limit curriculum breadth and global relevance (Holmgren & Eriksson, 2023).

Overall, significant variation exists in how nurses and midwives are educated across the world. So, although professional titles may be the same (such as Registered Nurse), while a graduate may be equipped to practise locally, they may not possess the skills needed to work elsewhere, to effectively consider how global issues might impact on their work or to influence global practice change. In fact, nurses and midwives educated in urban universities may not be well equipped even to work in rural or remote areas within their own countries. These issues are important to consider in ensuring universal access to appropriate health care.

Increasing emergence of global issues such as pandemics and disasters, climate change, artificial intelligence and nurse migration raises the question of whether there is a need to consider whether a more global approach to the development of curriculum standards is warranted (Holmgren & Eriksson, 2023). International organizations, such as the World Health Organization, the International Council of Nurses and the International Confederation of Midwives, already work across nursing and midwifery issues of global concern and produce guidelines for global implementation. However, the degree to which these are incorporated into localized nursing and midwifery curriculum standards is unclear. There is clearly a need to better understand how professional standards are being developed across the world, along with how they are ratified and implemented. The increasingly global nature of nursing and midwifery and their associated challenges suggests the need for a new global approach to the development of curriculum standards that reflect contemporary and globally relevant practice and promote global action (Holmgren & Eriksson, 2023).

## AUTHOR CONTRIBUTIONS

Ken Hok Man HO contributed to the conceptualization, project administration, writing—original draft, and writing—review and editing. Ho Yu CHENG, Lisa McKENNA and Daphne Sze Ki CHEUNG contributed to the conceptualization, writing—original draft, and writing—review and editing.

## FUNDING INFORMATION

The authors have no proprietary or commercial interest in any materials disclosed in this article.

## CONFLICT OF INTEREST STATEMENT

None.
